# Spatially-resolved metabolic profiling of living *Drosophila* in neurodegenerative conditions using ^1^H magic angle spinning NMR

**DOI:** 10.1038/s41598-020-66218-z

**Published:** 2020-06-11

**Authors:** Maxime Yon, Martine Decoville, Vincent Sarou-Kanian, Franck Fayon, Serge Birman

**Affiliations:** 1 CEMHTI UPR3079, CNRS, Université d’Orléans, F-45071 Orléans, France; 2 CBM UPR4301, CNRS, Université d’Orléans, F-45071 Orléans, France; 3 GCRN-LPC UMR8249, CNRS, ESPCI Paris, PSL Research University, F-75005 Paris, France

**Keywords:** Magnetic resonance imaging, Neurological models, Drosophila, NMR spectroscopy

## Abstract

*Drosophila* flies are versatile animal models for the study of gene mutations in neuronal pathologies. Their small size allows performing *in vivo* Magic Angle Spinning (MAS) experiments to obtain high-resolution ^1^H nuclear magnetic resonance (NMR) spectra. Here, we use spatially-resolved ^1^H high-resolution MAS NMR to investigate *in vivo* metabolite contents in different segments of the fly body. A comparative study of metabolic changes was performed for three neurodegenerative disorders: two cell-specific neuronal and glial models of Huntington disease (HD) and a model of glutamate excitotoxicity. It is shown that these pathologies are characterized by specific and sometimes anatomically localized variations in metabolite concentrations. In two cases, the modifications of ^1^H MAS NMR spectra localized in fly heads were significant enough to allow the creation of a predictive model.

## Introduction

Neurodegenerative disorders represent a critical challenge for general health as their incidence is in constant increase, in relation to population lifespan extension^1–3^. The identification of disease-specific biomarkers is a major issue in this field due to the difficulty in establishing early diagnosis and treatment before the onset of symptoms. In recent years, brain or biofluid metabolome profiling has become a promising tool to identify early diagnostic biomarkers of neurodegenerative diseases and potentially detect metabolic alterations that are common between different neurological conditions^4,5^. The high complexity and heterogeneity of the human metabolome justifies to perform such studies in parallel with simplified animal models, taking advantage of the recent adaptation of NMR techniques to invertebrate organisms such as *Caenorhabditis elegans* and *Drosophila melanogaster*^6–8^. Although the main occurrences of these diseases are sporadic, pathogenic mutations in genes playing a key role in pathogenesis have been identified, which has made it possible to transfer them to animal models for better understanding. Owing to its numerous advantages – well-known anatomy and diverse phenotypes, short life span, large number of progeny and high degree of conservation in fundamental biological pathways – the fruit fly *Drosophila melanogaster* has been a model of choice to study the function of these genes and the effects of their mutations on neuronal physiology^9–13^.

^1^H high-resolution magic angle spinning (HRMAS) nuclear magnetic resonance (NMR) spectroscopy has been widely used for *ex vivo* analysis of the metabolome in cell lines, animal models and human tissues^14–18^. Indeed, HRMAS allows the recovery of high-resolution ^1^H spectrum by narrowing the resonances which are broadened by magnetic field susceptibility and residual dipolar couplings present in inhomogeneous soft biological tissue in static conditions^19,20^. However, its application to living organisms remains challenging, particularly on mammals, as the spinning of the sample generates potentially lethal centrifugal effects^14^. Nevertheless, ^1^H HRMAS NMR has been successfully applied to *Drosophila*, by keeping the insects alive while spinning at about 2 to 3 kHz, thus enabling to compare metabolic profiles of wild-type and mutant flies^21,22^. Recently, our group improved the ^1^H HRMAS NMR analysis of *Drosophila* by obtaining the spectral signature of metabolites localized within a 1D ^1^H-density profile along the anteroposterior axis of the fly, thus enabling *in vivo* localized metabolic profiling^8^.

In the present study, this methodology is used to compare three different *Drosophila* models of neurodegeneration: two cell-specific models of Huntington disease (HD), in which a pathogenic fragment of the human Huntingtin (HTT) protein containing a pathogenic polyglutamine expansion was expressed either in neurons (NHD model)^23–29^, or in glial cells (GHD model)^25,26,30^, and a model of excitotoxicity-induced neurodegeneration based on the knock-down of the glutamate transporter Eaat1 in synapse-associated glia (GLU model)^31–36^. Glutamate excitotoxicity is an aggravating factor in several neurodegenerative conditions, including amyotrophic lateral sclerosis (ALS) and the consequences of stroke. It is shown that relative quantification of four main metabolites (fatty acids, glycerol, phosphocholine, and trehalose) by *in vivo* localized ^1^H HRMAS NMR spectroscopy enables to evidence significant metabolic differences between diseased and control flies in each part of the *Drosophila* body (head, thorax and abdomen). Interestingly, the observed changes in metabolite levels are found in several cases to be specific to the body segments. For the GLU and GHD models, we show that the relative changes of metabolite levels in fly heads allow discriminating diseased and control flies by partial least squares-discriminant analysis (PLS-DA).

## Materials and methods

### Drosophila stocks

Fly stocks were maintained at 22 °C on a standard medium (per liter: 90.25 g cornmeal, 82.5 g dry yeast, 10.75 g agar and 37.5 mL of a 10% solution of methyl-4-hydroxybenzoate in ethanol as an anti-fungal agent). Crosses were performed at 25 °C on this standard medium. The F1 progeny was collected each day and maintained at 25 °C on Nutri-Fly medium (Dominique Dutscher, Brumath, France). Only females were used for the HRMAS NMR experiments. The following *Drosophila* lines were used: *w*^1118^ as control, *elav-Gal4 (elav*^C155^)^37^, *Eaat1-Gal4*^32^, *UAS-Httex1p-Q93*^24^, and *UAS-Eaat1-IR*^31^. The neuronal and glial HD models were obtained by crossing *UAS-Httex1p-Q93* males to *elav-Gal4* or *Eaat1-Gal4* females, respectively (*elav* > *Httex1p-Q93* and *Eaat1* > *Httex1p-Q93* flies). The glutamate excitotoxicity model was obtained by crossing *UAS-Eaat1-IR* females to *Eaat1-Gal4* males (*Eaat1* > *Eaat1-IR* flies)^31^. Controls were the progeny of crosses between Gal4 drivers or UAS effectors with *w*^1118^.

### High-resolution MAS ^1^H NMR spectroscopic imaging

The ^1^H HRMAS NMR experiments on living *Drosophila* were performed essentially as previously described^8^, except that two flies were studied simultaneously. Two females were placed carefully in two separate compartments of a proton-free (PCTFE) cylindrical insert (top and bottom sides having a dedicated entrance) matching the size of the flies (~1 mm). The insert was then introduced in the center of the zirconia rotor so that the anteroposterior direction of the insects is aligned along the spinning axis. Anesthesia was obtained by maintaining the flies at 4 °C. The hardware and experimental parameters were identical to those described in Sarou-Kanian *et al*.^8^ the ^1^H HRMAS NMR spectra were recorded using a *Bruker AVANCE III HD* spectrometer operating at a magnetic field of 17.6 T (^1^H Larmor frequency of 750.13 MHz) with a 3.2 mm double-resonance MAS microimaging probe and a *Bruker Micro 2.5* gradient system (3 axes, 2.5 Gauss/cm/A, 60 A/axis). The acquisition spectral width was set to 10000 Hz and the 90° and 180° pulse durations were 12.5 µs and 25 µs, respectively. ^1^H chemical shifts were referenced using the resonance of the (CH_2_)_n_ of *Drosophila* fatty acids at 1.3 ppm as an internal reference (uncertainties of 0.02 ppm). Water suppression (WS) was achieved using a selective presaturation of the H_2_O resonance with a low-power pulse of 1 s. Data were processed with zero filling of twice the number of points. Exponential apodization with a 2-Hz line broadening was applied before Fourier transform. The spatial encoding was achieved with pulsed-field gradients applied along the MAS axis using a combination of the three orthogonal gradients (G_x_, G_y_, G_z_) such that $\vec{{{\rm{G}}}_{{\rm{MAS}}}}=(\vec{{G}_{x}}+\vec{{G}_{y}}+\vec{{G}_{z}})/\sqrt{3}$. The spectroscopic imaging along the anteroposterior direction of the flies was performed using a two-dimensional ^1^H HRMAS Chemical Shift Imaging (CSI) experiment^8^. The echo time was set to 10.6 ms and 64 gradient increments with 128 transients each were recorded with a recycle delay of 1.5 s. The duration of the G_MAS_ pulse was 400 µs with a maximum strength of 29.5 Gauss/cm leading to a field of view of 10 mm with a spatial resolution of 189 µm. The total experimental time was ~3.5 h. The rotor spinning frequency was 2630 Hz for the studies of 10-day-old *Eaat1* > *Httex1p-Q93* and *elav* > *Httex1p-Q93* flies. To avoid lethal damage to the fly abdomen, the rotor spinning frequency was reduced at 1280 Hz for the more fragile 10-day-old *Eaat1* > *Eaat1-IR* and 16-day-old *elav* > *Httex1P-Q93* flies. This reduction of the spinning rate ensures survival of the flies during the entire experiment. However, the reduction of spinning frequency leads to multiple spinning sidebands (satellite resonances at +/− the spinning frequency) and therefore a spinning sidebands suppression method (TOSS, TOtal Suppression of Sidebands^38^) was combined with the CSI sequence to obtain spatially-resolved spinning sideband-free ^1^H HRMAS spectra. The use of TOSS does not influence the quantification of the metabolites because only the center bands have been integrated in all the experiments. This partial integration does not take into account the sideband area and induces a slight underestimation of the total area (by approximately 20% at 1280 Hz). However, only the variations in sideband relative intensities influence the relative quantification. These variations, mainly due to magnetic field susceptibility fluctuations from one fly to another, are small and will be neglected in this study.

### Data processing

The 2D Chemical Shift Imaging (CSI) spectral maps were integrated over the three segments of the *Drosophila* body to obtain three localized 1D spectra corresponding to the head, thorax, and abdomen. The thorax zone was defined by the presence of β-alanine^8^ allowing a clear separation of the three segments. The baseline of the 1D spectrum was corrected on Matlab R2013b with a smoothing spline function calculated on the same baseline points for each series of spectra (containing control flies and *Httex1p-Q93-* or *Eaat1-IR*-expressing flies). The four main metabolites (fatty acids, glycerol, phosphocholine and trehalose) were quantified by integration and sum of their respective resonances. The saturated part and the unsaturated part of fatty acids molecules were integrated separately. Only the saturated part of the fatty acids molecules (CH_2_ and CH_3_) is represented since only few differences with the unsaturated part of fatty acids molecules could be observed. The metabolite levels were then normalized to 100 on the totality of the flies (control flies and *Httex1p-Q93-* or *Eaat1-IR-*expressing flies) and on the whole body of the flies to allow a comparison of the metabolite quantities in the head, thorax and abdomen, as well as between flies. The statistical meaning of the quantity differences of each metabolite between controls and *Httex1p-Q93-* or *Eaat1-IR-*expressing flies was assessed by one-way ANOVA and Tukey-Kramer tests performed with a confidence value of 0.05.

PLS-DA was also carried out on the metabolite resonances of the head profiles to discriminate between the control flies (merged into one group) and the corresponding diseased flies. The resonances corresponding to the main metabolites (fatty acids, glycerol, phosphocholine and trehalose) of the head 1D spectra were integrated. The integrals were normalized and mean-centered to give the same weight for each resonance and improve PLS-DA performances. The PLS-DA was performed with Matlab R2013b and the PLS toolbox (Eigenvector Research, Inc. Manson, WA 98831). The number of components was optimized to give the best Q² value with Venetian blinds cross-validation, with 10 splits and 1 sample per split.

## Results

**Figure 1:**
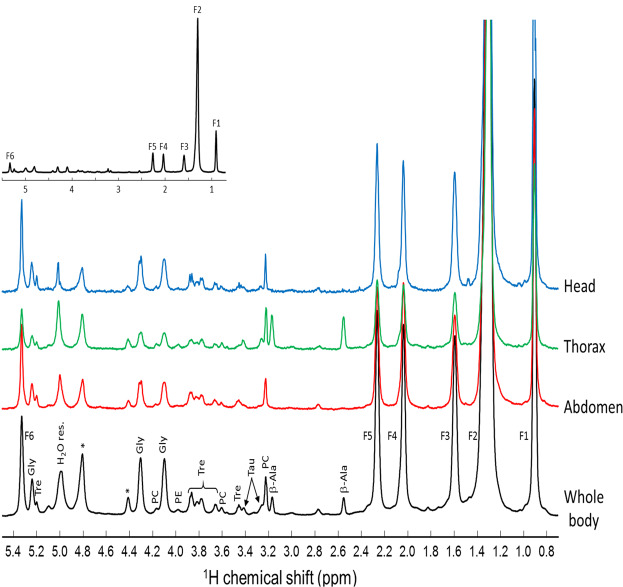
Representative ^1^H HRMAS NMR spectra obtained by various integration of a 2D Chemical Shift Imaging (CSI) map of a control *w*^1118^ *Drosophila* female in the 0.8–5.4 ppm range. The black spectrum is obtained by integration over the full-body while the blue, green and red spectra result from the specific integration of the head, thorax and abdomen regions. The 2D CSI map was recorded at a magnetic field of 17.6 T with a spinning frequency of 2630 Hz. F1, F2, F3, F4, F5, F6 correspond to fatty acid resonances of CH_3_, (CH_2_)_n_, CH_2_C-C=O, CH_2_C=, CH_2_C=O, and CH=CH, respectively. β-Ala: β-alanine; PC: phosphocholine; Tau: taurine; Tre: trehalose; PE: phosphoethanolamine; Gly: glycerol; *: spinning sidebands.

Representative spectra obtained by 2D Chemical Shift Imaging (CSI) of a control wild-type *w*^1118^ *Drosophila* are shown at two magnifications in Fig. 1. The black spectrum is obtained by integration over the full body of the fly, while the blue, green and red spectra are obtained by integration over the head, thorax and abdomen, respectively. They exhibit the ^1^H NMR spectral signature of typical major metabolites: fatty acids, phosphocholine, phosphoethanolamine, glycerol, trehalose, β-alanine, and taurine. The β-alanine is mainly present in the thorax and its resonances (2.53 and 3.19 ppm) are used to determine the boundaries of this segment^8^.

### Neuronal HD model

**Figure 2:**
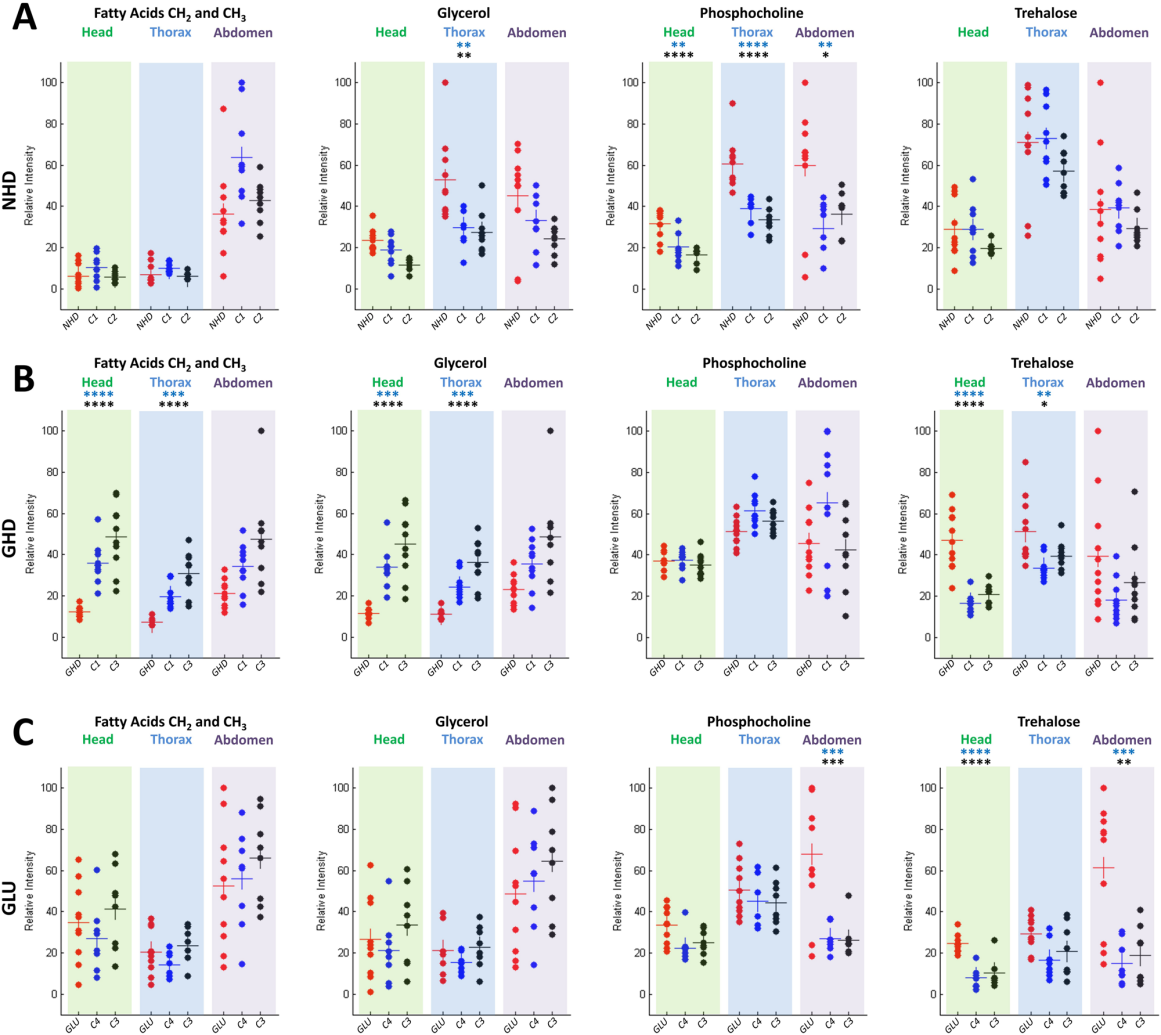
Comparison of relative metabolite concentrations in the head, thorax, and abdomen for the NHD (**A**), GHD (**B**) and GLU (**C**) neurodegenerative models. Each point represents the normalized quantity of a metabolite in a single fly. The NHD, GHD or GLU *Drosophila* are shown in red dots, the *UAS*/+ (*UAS-Httex1p-Q93* (C1) and *UAS-Eaat1-IR* (C4)) and *Gal4*/+ (*elav-Gal4* (C2) and *Eaat1-Gal4* (C3)) controls in blue and black dots, respectively. The horizontal bars indicate the mean metabolite quantity for each fly genotype. Stars above the graphs indicate *p* values of Tukey-Kramer tests when NHD, GHD or GLU flies were statistically different from the *Gal4* (black stars) and *UAS* (blue stars) controls. ****p < 0.0001, ***p < 0.001, **p < 0.01 and *p < 0.05.

The *Drosophila* neuronal HD model (NHD) recapitulates key symptoms of the disease: formation of HTT aggregates, progressive neuronal loss, locomotor defects, and early death. Here 10 and 16-day-old *elav* > *Httex1p-Q93* flies were studied, in which a fragment of human HTT carrying a pathogenic polyglutamine expansion of 93 residues was expressed pan-neuronally using the *elav-Gal4* driver^24,26–29^. For 10-day-old flies that are at a pre-symptomatic stage^26^, no obvious differences were detected between the control and NHD model spectra, except in the thorax where the phosphocholine signal (3.21 ppm) was found to be increased in the diseased flies (data provided as Supplementary Information, Fig. S1). The *p*-values for phosphocholine were 0.027 and 8.1 × 10^−4^ between NHD flies and the *UAS*/+ and *Gal4*/+ controls, respectively. For 16-day-old NHD flies (10 NHD, 8 *UAS-Httex1p-*Q93/+ (C1), and 8 *elav-*Gal4/+ (C2) controls), which exhibit a symptomatic stage corresponding approximately to the median lifespan in this model^26^, the comparison of the normalized spectra obtained for the NHD and control flies revealed several interesting metabolic features associated with neurodegeneration (Fig. 2A). The level of phosphocholine was found to increase significantly for NHD flies in the three anatomical parts of *Drosophila*, confirming the observation for the 10 days-old flies. The *p*-values for the head, thorax, and abdomen were respectively 1.8 × 10^−3^, 4.8 × 10^−5^, and 6.3 × 10^−3^ between NHD and C1 controls, and 6.2 × 10^−5^, 1.8 × 10^−6^ and 3.6 × 10^−2^ between NHD and C2 controls. The level of glycerol (4.1, 4.31 and 5.24 ppm) also significantly increased in NHD flies, specifically in the thorax (*p*-values 4.7 × 10^−3^ and 1.9 × 10^−3^ between NHD and the C1 and C2 controls, respectively). No significant differences between NHD and control flies were found for fatty acids and trehalose in the three body regions.

### Glial HD model

The expression of mutant human HTT in brain glial cells of *Drosophila* also leads to the formation of protein aggregates, locomotor defects, and early death, like in the NHD model, but it has been suggested that mutant HTT (mHTT) activates distinct pathways of toxicity in fly neurons and glia^25,26,30^. 10-day-old glial HD model (GHD) flies expressing *Httex1p-Q93* in astrocyte-like glial cells with the *Eaat1-Gal4* driver^25^ were analyzed and compared to control flies (11 GHD, 10 *UAS-Httex1p-*Q93/+ (C1), and 10 *Eaat1-*Gal4/+ (C3) controls). The comparison of the average spectra revealed that the levels of glycerol, trehalose and fatty acids were different between controls and GHD flies in the head and the thorax (Fig. 2B). Statistical analysis revealed a strong decrease in fatty acids concentration in the head (*p* values: 7.5   10^−5^ and 5.8   10^−8^ for comparisons between GHD flies and C1 and C3 controls, respectively) and the thorax (*p* values: 5.9   10^−4^ and 2.4   10^−8^ for the same comparisons). The glycerol peak was also significantly decreased in the head (*p* values: 1.3   10^−4^ and 2.6   10^−7^) and the thorax (*p* values: 7.6   10^−4^ and 4.1   10^−8^). In contrast, a significant increase in trehalose content was observed both in the head (*p* values: 7.8   10^−8^ and 1.2   10^−6^) and thorax (*p* values: 2   10^−3^ and 3.9   10^−2^). No significant difference between GHD and control flies was found in the abdomen for all these metabolites (Fig. 2B).

### Glutamate excitotoxicity model

Knocking down of the glutamate transporter Eaat1 in *Drosophila* prevents extracellular glutamate buffering in the nervous system and triggers a variety of phenotypes, including excitotoxicity-induced neurodegeneration^31–36^. 10-day-old *Eaat1* > *Eaat1-IR* flies (10 GLU, 9 *Eaat1-*Gal4/+ (C3), and 9 *UAS-Eaat1-IR*/+ (C4) controls), in which *Eaat1* was inactivated by RNAi in the glial cells that normally express this transporter (GLU model)^31^ were analyzed in this study. The comparison of the GLU model and control flies revealed no difference for fatty acids and glycerol concentrations in the three body segments (Fig. 2C). In contrast, trehalose content was significantly increased in the head (*p* values: 3.8   10^−5^ and 4.5   10^−6^ between GLU flies and the C3 and C4 controls, respectively) and the abdomen (*p* values: 1.1   10^−3^ and 4.5   10^−4^, for similar comparisons). Unexpectedly a strong increase in phosphocholine content was also found selectively in the abdomen of the GLU flies. In agreement with these ^1^H NMR results, a biochemical dosage of trehalose performed on the whole fly indicated a significant increase in trehalose level in the GLU model compared to controls (data provided as Supplementary Information, Fig. S2).

### Distinction between control and diseased fly head 1D spectra using a multivariate test

Focusing on the head region, we showed that univariate analysis on the main peaks enabled to identify metabolites that varied significantly between diseased and control *Drosophila*. To confirm these results and allow a discrimination directly based on the head resonance integrals, we used partial least squares discriminant analysis (PLS-DA). This supervised multivariate analysis allows distinction between the classes (diseased or control flies) by projecting the variable variance in a space of fewer dimensions described by the Latent Variables (LVs). The first obtained LV (LV1) expresses the correlation between the variables and the classes and is thus optimized to show the inter-classes (control/diseased) differences. The next LVs (LV2…) also contain the compensation correlations, which might not be primarily related to the classes and are more difficult to interpret^39^. The PLS-DA also allows a quantification of the discriminative power of each variable through their cross-validated multiple correlation coefficient values (Q²) in the first LV^39^ and permits to build a predictive model.

**Figure 3:**
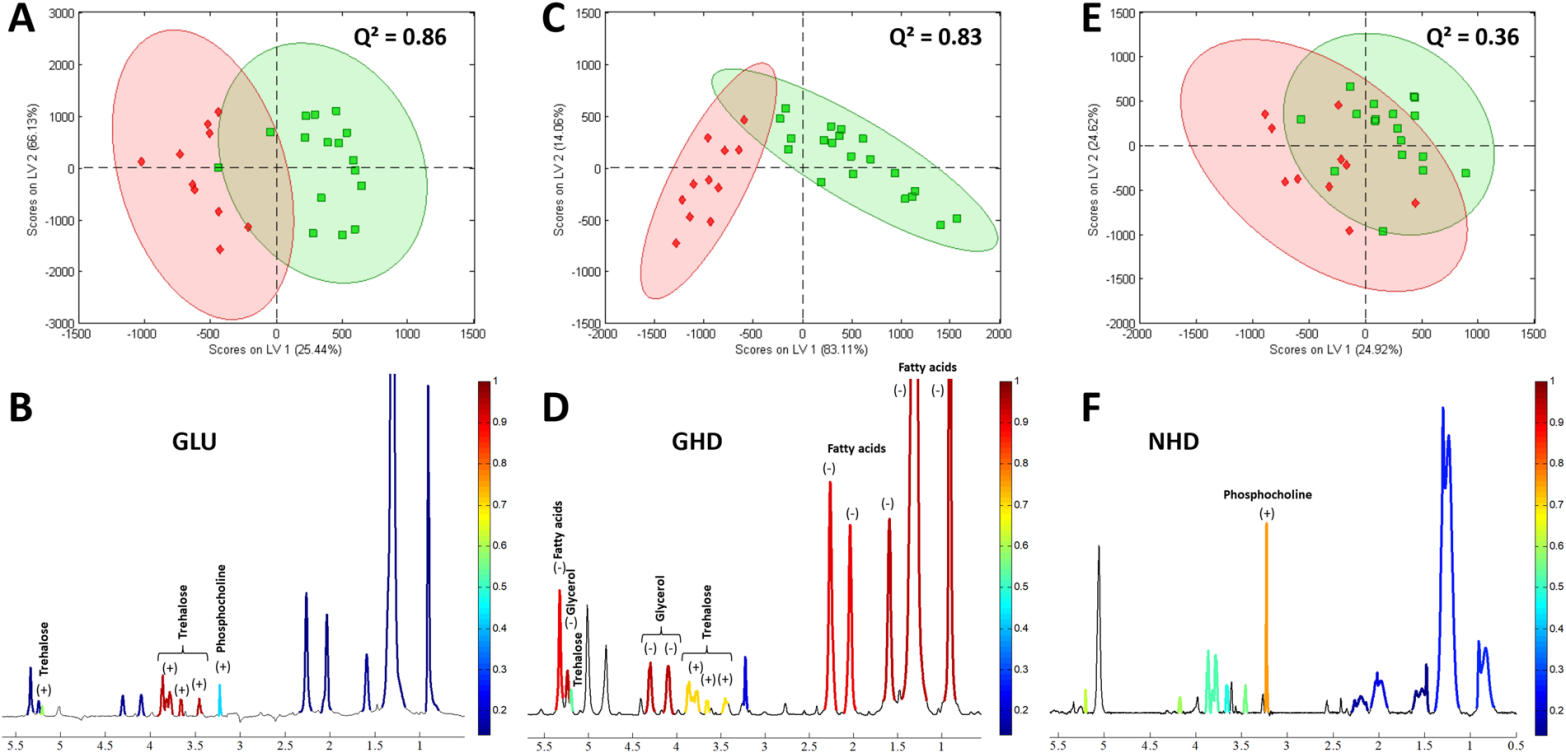
PLS-DA on the main ^1^H resonances localized in fly head. (**A,C,E**) PLS-DA score plots derived from localized 1D ^1^H MAS NMR spectra of *Drosophila* heads. Green solid squares: control flies; red solid diamonds: GLU (**A**), GHD (**C**) or NHD (**E**) flies. The first and second Latent Variables are denoted LV1 and LV2, respectively. Each point represents a single fly. 95% confidence ellipses were drawn. A clear separation is revealed across the LV1 components of control flies and GLU or GHD, but not NHD flies. (**B,D,F**) Mean spectra derived from the whole series of 1D spectra localized in fly head for GLU (**B**), GHD (**D**) or NHD (**F**) flies. The colored parts show the integrated areas. The color scales indicate the Q² values obtained with the first latent variable of the PLS-DA analysis, red corresponding to the highest significance and blue to no significance. Positive (+) and negative (−) signs indicate an increase or a decrease in metabolite concentrations in the GLU, GHD or NHD expressing groups compared to controls, respectively.

The results of this analysis are presented in Fig. 3. For each of the groups, the resonances of a single metabolite always exhibit a roughly constant Q², as expected since their areas vary simultaneously, except for the resonance of the trehalose at 5.2 ppm, which is randomly affected by water suppression. The Q² value is also roughly constant between the ^1^H resonances attributed to the saturated and the unsaturated part of the fatty acids, indicating a steady proportion of these two functions in the fatty acids chains. In the case of the GLU model, the PLS-DA allowed discrimination between *Eaat1-IR*-expressing flies and controls with a value of Q² = 0.86, indicating a reliable predictive model (Fig. 3A). The separation (carried out by the first PLS-DA component) represents 25% of the total variance of the system and is mainly due to the trehalose resonances, as shown in red in the mean spectrum (Fig. 3B), although the phosphocholine also participates slightly. The spectrum analysis on the GHD model also allowed discrimination between the two groups (controls and *Httex1p-Q93*-expressing flies) with a Q^2^ value = 0.83 (Fig. 3C). The separation (carried out by the first PLS-DA component) represents 83% of the total variance of the system and is mainly related to the fatty acids and glycerol contents, and to a lesser extent to trehalose (Fig. 3D). In contrast, a valid PLS-DA model could not be established with the NHD flies expressing *Httex1p-Q93* in neurons (Q² = 0.36) (Fig. 3E), in spite of the prominent increase in phosphocholine (Fig. 3F).

## Discussion

Our results show that the localized relative quantification of four main metabolites (fatty acids, phosphocholine, glycerol, and trehalose) using *in vivo* spatially-resolved HRMAS ^1^H NMR spectroscopy is sufficient to distinguish metabolic changes specific to three previously established *Drosophila* models of neurodegeneration, i.e. neuronal and glial models of HD (NHD and GHD) and a model of glutamate excitotoxicity (GLU).

First, we observed that *Drosophila* expressing *Httex1p-Q93* in neuronal cells (NHD model) are mainly characterized by an increase in phosphocholine concentration. Similar results have already been observed in HD patients using magnetic resonance imaging (MRI)^40,41^. Other studies using the mouse R6/2 HD model have also revealed an increase in phosphocholine^42–44^. Phosphocholine molecules are precursors of phosphatidylcholines that are major components of eukaryotic cellular membranes (in particular neuronal membranes) and of mitochondrial membranes^45–47^. They participate in the formation of new membranes and synapses and it has been shown that the administration of phosphatide precursors enhances synapse formation^48–50^. It was also demonstrated that choline metabolism is dependent on the mitochondrial electron transport chain^51^. The increase in phosphocholine content observed for the NHD model could thus result either from neuronal membrane degeneration or from mitochondrial impairment associated with HD^52^.

Quite interestingly, we have found that the expression of *Httex1p-Q93* in glial cells (GHD model) alters metabolism totally differently compared to the NHD model, indicating that expressing mHTT in neurons or glia triggers pathologies that lead to brain failure through different pathways of toxicity, as previously suggested^25,26,30^. Indeed, in the GHD model, no change in phosphocholine content was observed, but the trehalose level was higher and lipids and glycerol levels were lower. Variations of these metabolite concentrations may reflect an alteration in energy metabolism. In *Drosophila* and other insects, trehalose is the major circulating metabolite supplying energy to cells^53^. It is synthesized in the fat body^54^, an organ comparable to mammalian liver and adipose tissue, and Volkenhoff *et al*.^55^ have shown that trehalose is transported into the brain by the blood-brain-barrier perineurial glia, in which it is converted to alanine and lactate that are used to fuel neurons. Glycerol, fatty acids, glutamine or glutamate may be used as an alternative energy source, in particular under pathological conditions^56–58^. A lower glycerol level may then suggest that the transformation of glycerol into glucose and then trehalose is favored in the GHD model.

The GLU model, obtained by inactivation of the glutamate transporter *Eaat1* in glial cells is characterized by an increase in trehalose level as in the GHD model, as well as an increase in phosphocholine content in the abdomen as in the NHD model. This is interesting as we previously demonstrated that the expression of mHTT in glial cells leads to a decrease in *Eaat1* expression in *Drosophila*^25^. Sibson *et al*.^59^ have studied the relationship between glucose metabolism and glutamatergic neuron activity. They have shown that uptake of one glutamate molecule by astrocytes and its conversion into glutamine requires two ATP molecules that are produced by glucose metabolism. In the GHD and GLU models, glutamate uptake is impaired because of the decrease in *Eaat1* expression. Consequently, glucose needs would be decreased and this, in turn, could in part explain the increase in trehalose level.

Furthermore, trehalose is known to have potent neuroprotective properties in various mammalian models of neurodegenerative diseases^60–65^, which makes it a drug with promising therapeutic potential for humans^66^. Although it was initially thought that trehalose acts by inducing directly autophagy in neurons, thus promoting the clearance of protein aggregates^60,62^, recent *in vitro* studies have challenged this interpretation and suggested that its established effect on neuronal autophagy in *in vivo* models could actually be indirect^67,68^. Because trehalose is neuroprotective, the high increase in trehalose level we observed here in the GHD and GLU models could correspond to a defense reaction of the organism in these specific pathological conditions. Although it has been shown that trehalose protects *Drosophila* from hypoxic and anoxic injury^69^, no evidence that trehalose is protective in fly models of neurodegeneration was provided to date to our knowledge. Conducting such studies in flies could be useful to decipher genetically the neuroprotective mechanisms promoted by trehalose in mammals.

A major strength of the approach used here relies on the *in vivo* measurement of metabolite concentrations in the different anatomical parts of the fly which makes possible to know if the alteration of metabolism is a systemic effect or limited to a specific organ. We observed that when mHTT was expressed in neuronal cells, the increase in phosphocholine content concerned the whole body, suggesting a systemic effect. In contrast, the alteration of metabolism concerned only specific parts of the fly body in the glial models. For the GHD model, metabolite concentration variations concern only the head and thorax regions, suggesting a selective localization of the defects in the central nervous system. In the case of the GLU model, modifications are observed in the head and the abdomen. The significant increase in phosphocholine and trehalose levels in the abdomen for the GLU model was unexpected and may suggest that intestinal defects and perturbation in the gut-brain axis play an important role in this specific pathology. Interestingly, this would indicate that metabolic changes are not restricted to the central nervous system in fly neurodegenerative disease models, but can be detected in some cases in the abdomen as well.

For both GLU and GHD models, the PLS-DA on the main resonances of the 1D spectra led to meaningful models discriminating the control and diseased groups with Q² of respectively 0.86 and 0.83. The separation of these two groups is mainly due to the metabolites identified in the univariate analysis. However, the control and diseased groups widely overlap for the NHD model, making the construction of a correct predictive model impossible. This analysis still identifies phosphocholine as a discriminating metabolite, as shown by the univariate test. The possibility to create a predictive model directly based on the 1D spectra localized in the head paves the way for the distinction of similar pathologies in mammalian models by localized brain spectroscopy, for which the ^1^H resonances of fatty acids and of the main metabolites with a chemical shift ranging between 0 and 4 ppm can be resolved without magic angle spinning^70^.

## Conclusions

In conclusion, these results demonstrate that the use of spatially-resolved ^1^H high-resolution magic angle spinning NMR can provide essential information about metabolic perturbations at the scale of the body segments in various *Drosophila* disease models. These results pave the way for the discrimination of neurodegenerative diseases by localized ^1^H NMR spectroscopy of the brain in mammalian models where some of the observed metabolites can also be quantified.

## Supplementary information

Supplementary information.
