# Supplementary material for: Spatially-resolved metabolic profiling of living Drosophila in neurodegenerative conditions using 1H magic angle spinning NMR
Source: Sci Rep. 2020 Jun 11;10:9516. doi: 10.1038/s41598-020-66218-z (PMC7289880; doi:10.1038/s41598-020-66218-z)
Supplement: Supplementary file 1 — Supplementary information. [file 41598_2020_66218_MOESM1_ESM.docx]

**Supplementary material**

**Spatially-resolved metabolic profiling of living *Drosophila* in neurodegenerative conditions using ^1^H magic angle spinning NMR**

Maxime Yon^1^, Martine Decoville^2^, Vincent Sarou-Kanian^1*^, Franck Fayon^1^ & Serge Birman^3^

^1^CNRS, CEMHTI UPR3079, Université d’Orléans, F-45071, Orléans, France.

^2^CNRS, CBM UPR4301, Université d’Orléans, F-45071, Orléans, France.

^3^CNRS, GCRN-LPC UMR8249, ESPCI Paris, PSL Research University, F-75005, Paris, France.


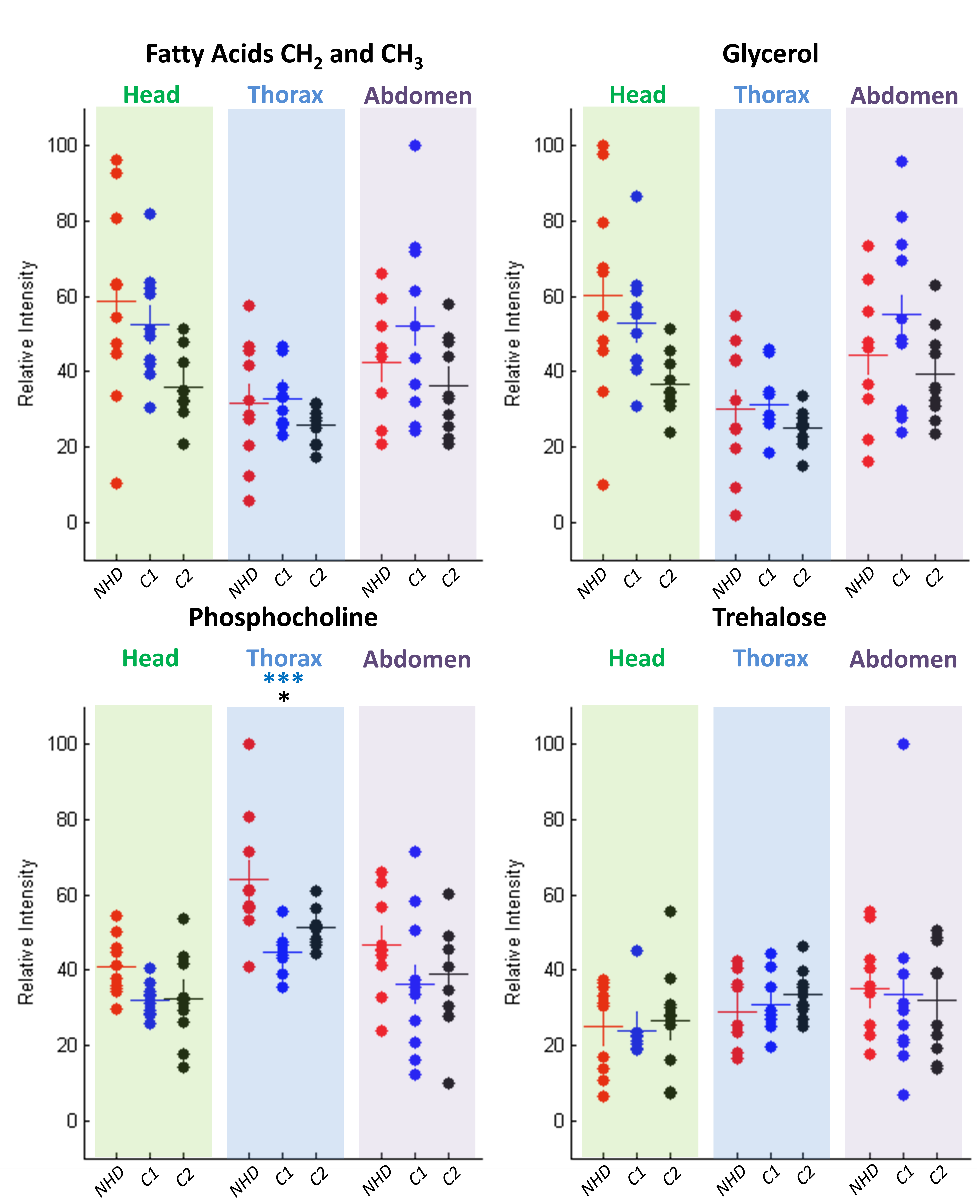


**Fig. S1.** Comparison of relative metabolite changes in the head, thorax, and abdomen of 10-day-old NHD model flies. Each point represents the normalized quantity of a metabolite in a single fly. The NHD *Drosophila* are shown in red points, the *UAS-Httex1p-Q93*/+ (C1) controls in blue and the *elav-Gal4*/+ (C2) controls in black points. The horizontal bars indicate the mean metabolite quantity for each fly genotype. Stars above the graphs indicate *p* values of Tukey-Kramer tests when NHD flies were statistically different from the *Gal4* (black stars) and *UAS* (blue stars) controls: ***p<0.001 and *p<0.05.


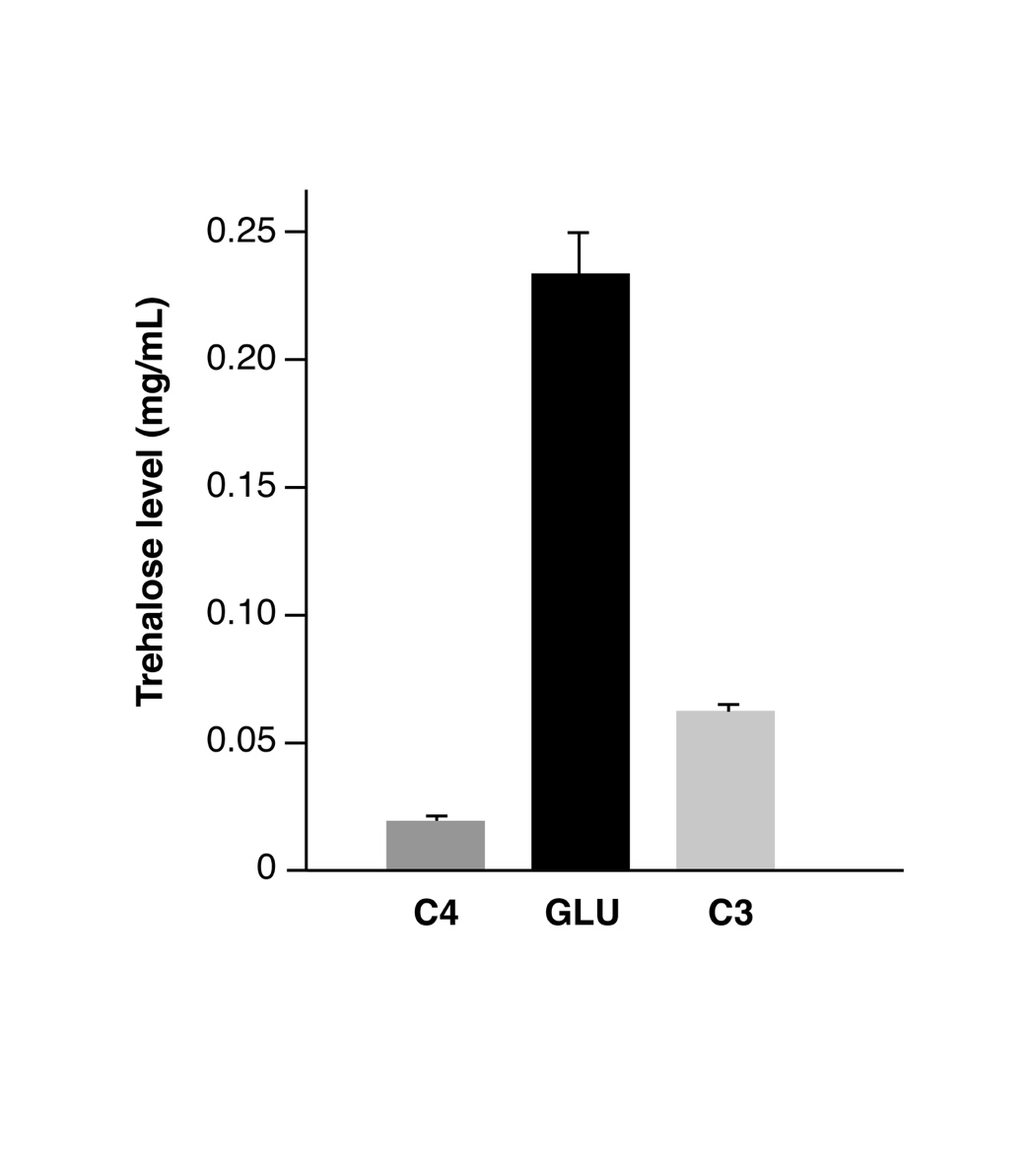


**Fig. S2.** Biochemical assay of trehalose in 10-day-old GLU model flies. Whole fly trehalose level was determined by using the Glucose (HK) Assay Kit (Sigma-Aldrich, cat. GAHK20-1KT) as described by Broughton et al. (2005). The GLU model is compared to *Eaat1-*Gal4/+ (C3) and *UAS-Eaat1-IR*/+ (C4) controls. Before incubating the extract from 10 flies of each genotype with trehalase (Sigma-Aldrich, cat. T8778) overnight at 37°C, a first glucose assay was performed. The amount of glucose was measured again after the incubation. The level of trehalose corresponds to the difference between the two assays. Bars indicate standard deviations.
